# Supplementary material for: Content-rich biological network constructed by mining PubMed abstracts
Source: BMC Bioinformatics. 2004 Oct 8;5:147. doi: 10.1186/1471-2105-5-147 (PMC528731; doi:10.1186/1471-2105-5-147)
Supplement: Additional File 2 — The original results of the above study (non-essential files are deleted to keep the file size under the limit set by BMC bioinformatics). [file 1471-2105-5-147-S2.bz2 › chilibotAdditionalFile2/dip20/356ID9062194E4998/html/IL1R1_IL1RN.html]

 


 **IL1R1** and **IL1RN** 
  
Found 1322 abstracts in PubMed, retrieved 20.  
 

 What does Google say? 
 PDF only 
| .edu only 

---

**Interactive relationship** (e.g. stimulation, inhibition, etc)

**Non-interactive relationship** (e.g. studied together, co-existance, homology, etc.)

- The authors wished to evaluate whether serum levels of interleukin 1 receptor  [ **IL1R1** ]  agonist IL 1Ra  [ **IL1RN** ]  and tumor necrosis factor alpha TNF alpha are elevated in children with LCH and decrease after chemotherapy.PATIENTS AND METHODSCirculating levels of IL 1Ra  [ **IL1RN** ]  and TNF alpha were measured in 23 and 8 children with LCH, respectively, and 7 pediatric controls using commercially available ELISA kits.  Ref: 12794527 J Pediatr Hematol Oncol, 2003
- When recombinant IL1ra  [ **IL1RN** ]   [ **IL1R1** ]  was titrated into the assay, phagocytosis of the opsonized RBC was significantly inhibited p=0.002.  Ref: 12829590 Blood< MedlineTA>Blood, 2003
- In this study, we injected recombinant adeno associated virus rAAV vectors expressing the interleukin 1 receptor  [ **IL1R1** ]  antagonist rAAV IL 1ra  [ **IL1RN** ]  into the cortex of rats experiencing transient cerebral ischemia.  Ref: 12858036 Neuroreport, 2003
- Cytokines interleukin 8 IL 8, interleukin 10 IL 10, interleukin 1 receptor  [ **IL1R1** ]  antagonist IL 1ra  [ **IL1RN** ]  and tumour necrosis factor soluble receptor 2 TNFsr2 and the leucocyte adhesion molecules L selectin, HLA DR, CD18 and CD11b were determined.  Ref: 12842763 Cytokine, 2003
- Interleukin 1 receptor  [ **IL1R1** ]  antagonist IL 1ra  [ **IL1RN** ]  is an important anti inflammatory cytokine that blocks all known actions of IL 1 and markedly protects against experimentally induced ischemic, excitotoxic, and traumatic brain insults.  Ref: 12878687 J Neurosci, 2003
- Interleukin 1 receptor  [ **IL1R1** ]  antagonist IL 1RA  [ **IL1RN** ]  polymorphism in women with cervical cancer.  Ref: 12820354 Anticancer Res< MedlineTA>Anticancer Res, 2003
- The decline in phagocytosis was correlated with a significant production of IL1 receptor antagonist IL1ra  [ **IL1RN** ]   [ **IL1R1** ]  by both monocytes p=0.036 and granulocytes p=0.0002 within 4 hours.  Ref: 12829590 Blood< MedlineTA>Blood, 2003
- Median clearance values ranged between 25 and 54 ml min for interleukin 1 receptor  [ **IL1R1** ]  antagonist IL 1ra  [ **IL1RN** ] , 23 and 42 ml min for IL 6 and 15 and 28 ml min for TNF alpha.  Ref: 12808174 Nephrol Dial Transplant< MedlineTA>Nephrol Dial Transplant, 2003
- Taken together, these results suggest that at least one mechanism of action of anti D is via the production of the anti inflammatory cytokine IL1ra  [ **IL1RN** ]   [ **IL1R1** ]  which can negatively regulate the ability of leukocytes to phagocytose opsonized cells.  Ref: 12829590 Blood< MedlineTA>Blood, 2003
- Using colonic explant cultures from 132 IBD and control subjects, we examined tumour necrosis factor alpha TNF alpha, interleukin IL 1 and interleukin 1 receptor  [ **IL1R1** ]  antagonist IL 1RA  [ **IL1RN** ]  production in vitro in response to bacterial activators.  Ref: 12823284 Clin Exp Immunol, 2003
- RESULTS We demonstrated that interleukin 1beta IL 1beta, interleukin 1 receptor  [ **IL1R1** ]  2 IL 1R2 and interleukin 1 receptor  [ **IL1R1** ]  antagonist IL 1RA  [ **IL1RN** ]  genes are expressed in equine granulosa cells.  Ref: 12803652 Reprod Biol Endocrinol< MedlineTA>Reprod Biol Endocrinol, 2003
- All subjects underwent plasma insulin, glucose, interleukin 6 IL 6, interleukin 10 IL 10, interleukin 1beta IL 1beta, interleukin 1 receptor  [ **IL1R1** ]  antagonist IL 1ra  [ **IL1RN** ] , C reactive protein, and tumor necrosis factor alpha TNF alpha levels determination.  Ref: 12850386 Am J Hypertens, 2003
